# Advance in Managing Indoor Cat Allergen Proteins: Molecular Insights, Detection, and Control

**DOI:** 10.3390/ijms262210913

**Published:** 2025-11-11

**Authors:** Yuxin Jiang, Xinya Tian, Xiaoxin Fu, Baichuan Ma, Zhenlong Wang, Bing Han, Hui Tao, Jinquan Wang, Xiumin Wang

**Affiliations:** 1 Institute of Feed Research, Chinese Academy of Agricultural Sciences, Beijing 100081, China; 821012450527@caas.cn (Y.J.); 82101232041@caas.cn (X.T.); fuxiaoxin2000@outlook.com (X.F.); 821012530042@caas.cn (B.M.); wangzhenlong02@caas.cn (Z.W.); hanbing02@caas.cn (B.H.); taohui@caas.cn (H.T.); jinquanwang@caas.cn (J.W.); 2 Key Laboratory of Feed Biotechnology, Ministry of Agriculture and Rural Affairs, Beijing 100081, China

**Keywords:** allergy, indoor cat allergen, detection, influencing factor, treatment

## Abstract

Indoor cat allergens, particularly the major allergen Fel d 1 protein, represent significant environmental triggers for allergic rhinitis, asthma, and other immune-related disorders in humans. With the continuous global increase in pet ownership, cat allergen proteins are prevalent in diverse settings and can even be transmitted to pet-free locations via clothing and animal fur, thereby posing health risks to sensitized individuals. This review systematically summarizes the molecular characteristics, distribution patterns, and mechanisms of human sensitization to indoor cat allergen proteins. It focuses on a comparative analysis of the principles, sensitivity, and application of commonly used immunological methods (such as various modified ELISAs, immunoblotting, and high-throughput multiplex detection technologies) alongside emerging real-time sensing platforms (including QCM, SAW, and LIF). Furthermore, this review summarizes key factors affecting indoor allergen concentrations, such as cat characteristics, architectural environments, human activities, and spatiotemporal variations. It also evaluates the efficacy and limitations of current allergy control strategies, covering source control (e.g., gene editing, immunomodulation), environmental management (e.g., air filtration), and medical treatments (e.g., allergen immunotherapy), and discusses future prospects. This review aims to offer a scientific foundation and systematic reference for the detection, control, and public health protection related to indoor cat allergens.

## 1. Introduction

Allergic reactions are abnormal immune responses to typically environmental antigens, such as pollen, dust mites, microorganisms, and animal dander [1]. These conditions exhibit significant individual variability and include diverse clinical manifestations, such as asthma, allergic rhinoconjunctivitis, sinusitis, atopic dermatitis, angioedema, and urticaria. These symptoms may present in isolation or co-occur with other symptoms [2,3]. Asthma and atopic dermatitis, as classic allergic diseases, have seen rising global prevalence since the 1990s. Concurrently, the incidence of allergic rhinitis and asthma continues to rise [4]. In South Korea, for instance, allergic rhinitis affects approximately 37.6% of the population [5], while certain countries report asthma diagnosis rates as high as 15–20% [6]. According to the World Health Organization (WHO), the global number of asthma cases is expected to rise to 100 million by 2025 [7]. Modeling studies suggest that by 2050, over 15% of the population in key regions will be affected, amounting to an estimated 423 million cases globally [8,9,10,11].

In recent decades, allergies to furry animals have grown increasingly prevalent worldwide [7]. Epidemiological studies globally confirm that cat and dog allergens are major triggers of allergic sensitization and clinical symptoms [12]. In North America, sensitization rates to these animals can exceed 50% (Table 1). A nationwide allergy survey in China emphasized the critical role of assessing patients’ specific antibody levels for accurate allergen identification, reporting that sensitization rates to cats and dogs rose from 1.33% and 0.83% in 2009 to 15.47% and 10.51% in 2021, respectively (Table 1) [13]. This increasing sensitization coincides with expanding pet ownership, indicating growing population exposure to these allergens. A large international survey across 22 countries involving over 27,000 participants revealed that 57% of households owned at least one pet, with dogs (33%) and cats (23%) being the most common [14]. In China, the number of dogs and cats kept in urban households increased from 99.15 million in 2019 to 120 million in 2023 [15,16]. This sustained growth in pet ownership suggests that environmental levels of cat and dog allergens will continue to increase. Pet owners can inadvertently transport allergens on clothing and hair [17], facilitating their transfer into various public spaces, such as schools, workplaces, or recreational facilities [18]. Even in households without pets, detectable levels of cat and dog allergens are often present, particularly in communities where pet ownership is common [19].

Currently, convenient and effective control and intervention methods for managing health risks associated with indoor cat allergens remain insufficient. One report indicates that Fel d 1, the dominant cat allergen, can remain in indoor air for extended periods, up to 6–9 months, after the removal of the cat from the environment [21]. Existing strategies can be categorized into four types. First, environmental management interventions aim to reduce airborne allergen levels through cleaning, ventilation, and high-efficiency filtration. However, their efficacy is often limited by the strong adhesive properties and aerodynamic behavior of allergens [22]. Second, animal-source interventions, such as administering products containing anti-Fel d 1 specific IgY target the reduction in active allergen secretion from cat saliva and dander at the source [23]. Third, symptom-oriented drug therapy involves the use of antihistamines and nasal corticosteroids to alleviate body’s reactions after an allergic response occurs [24]. Fourth, allergen immunotherapy induces immune tolerance through prolonged, gradually increased exposure to allergens; however, its application is limited by extended treatment periods and heterogeneous patient responses [25,26].

In this study, a comprehensive literature review was performed on cat allergens, with systematic evaluation of their distribution characteristics, transmission pathways, and factors influencing exposure levels. Furthermore, we analyzed the principles, applicable scenarios, and utility of various detection technologies for allergen monitoring and control, aiming to propose improved strategies for the accurate detection and effective management of indoor cat allergen proteins. Promising future research directions are also discussed.

## 2. Allergens from Cats

### 2.1. Molecular Insights of the Major Cat Allergen Fel d 1

Fel d 1 represents the dominant allergen in domestic cats [27]. At the molecular level, it is classified as a secretory protein belonging to the hemoglobin family, rather than the lipid carrier protein family [28,29]. This glycoprotein has a molecular weight of approximately 35–38 kDa [30]. Its quaternary structure consists of two identical homodimers that link noncovalently to form a tetramer, with each homodimer weighing approximately 18–19 kDa [31]. Although the biological function of Fel d 1 remains unclear, some studies reveal its structural homology with uterine hemoglobin, suggesting a potential role in skin barrier protection [32]. Alternatively, it may be involved in the transport of lipid molecules, such as steroids, hormones, or pheromones [33].

**Figure 1:**
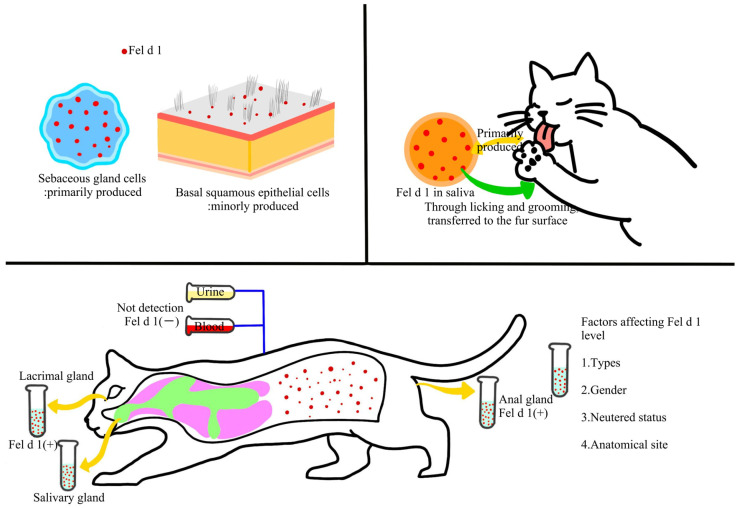
Production, secretion, and transfer of allergen proteins (e.g., Fel d 1, Fel d 4, etc.) in cats.

Regarding its role in sensitization, Fel d 1 is the major trigger of allergic responses to cats. Specific IgE antibodies against Fel d 1 are detectable in the serum of 80–95% of individuals allergic to cats [34], accounting for 60–90% of the total IgE response directed against cat allergens in sensitized patients [35,36]. The allergen is primarily produced in sebaceous glands and squamous epithelial cells and transferred onto the fur through grooming and licking behaviors [37]. Skin biopsy studies further indicate that Fel d 1 is mainly secreted by sebaceous cells, with minor production in basal squamous epithelial cells, and is predominantly deposited in the epidermis and on the hair surface [38,39]. It is also found in salivary glands, perianal glands, and lacrimal glands, but is absent in urine or serum [40,41,42]. Notably, Fel d 1 levels are influenced by several factors, including cat species, sex, sterilization status, and anatomical sites (Figure 1) [43,44,45,46].

### 2.2. Other Cat Allergens and Their Cross-Reactivity

Cats present a complex reservoir of allergen proteins. Besides the predominant allergen Fel d 1, other protein components contribute to sensitization [47]. The WHO/International Union of Immunological Societies (IUIS) has officially classified eight allergens from the domestic cat (*Felis domesticus*), designated as Fel d 1 to Fel d 8 [48]. These proteins exhibit considerable diversity in both structure and biological function: Fel d 1 is a uteroglobin-like molecule, Fel d 2 is serum albumin, Fel d 3 is cystatin [49], Fel d 4 and Fel d 7 belong to the lipocalin family [50], Fel d 5 and Fel d 6 are immunoglobulins, and Fel d 8 is a latherin-like protein [51]. Among these, Fel d 3, Fel d 4, and Fel d 7 are particularly notable, each capable of triggering IgE-mediated responses in more than 50% of individuals allergic to cats [52]. Notably, many of these minor allergens display high structural conservation with homologous proteins from other species, forming a molecular basis for cross-reactivity [53,54]. For instance, Fel d 2 (serum albumin) shares high sequence identity with Can f 3 from dogs (Table 2) [55], which can lead to cross-recognition of serum albumins from cats, dogs, and even other mammals (such as horses, mice, and cattle) [56]. Similarly, Fel d 4 and Fel d 7, as lipocalins, demonstrate functional and structural parallels with canine allergens Can f 1, Can f 2, and Can f 4 [57]; Fel d 4 shows high sequence similarity to Can f 6 from dogs (Table 2) [58]. These extensive cross-reactivities, combined with individual variations in sensitization, contribute to the heterogeneous clinical manifestations of allergy to cats and support the use of component-resolved diagnosis (CRD) for personalized treatment [59].

### 2.3. Distribution and Transmission of Cat Allergens in Indoor Environments

The particle size distribution of the major cat allergen Fel d 1 in indoor environments is influenced by multiple factors. Some studies indicate that Fel d 1 is predominantly carried on fine particles (≤5 μm) [60], which can remain suspended in the air for extended periods; however, others report a stronger association with larger particles (>4 μm) [61,62]. Despite these differences, a stable fraction of Fel d 1 bound to small particles has been detected across studies. Environmental disturbances such as air movement or human activity can resuspend large particles, thereby altering the overall size distribution [63]. The levels of Fel d 1 in residences with cats are significantly higher than those without cats [64]. Nevertheless, considerable levels of this allergen are still measurable in cat-free environments [13,19,65]. Cat allergens have also been detected in schools [66], offices [67], and public transportation systems [68], often at levels sufficient to trigger allergic symptoms in sensitized individuals. Primary sources of Fel d 1 in these settings include allergen-contaminated clothing [69] and the cross-environmental transport of cat hair. Clothing serves as a vehicle for transporting cat allergens into cat-free environments, where they subsequently settle on surfaces and become suspended in the air [70]. Similarly, human hair represents another significant medium for the transfer and accumulation of cat allergens in public areas [71]. This mode of transmission explains why cat allergens can still be detected even in settings where strict avoidance measures are implemented [72].

### 2.4. Exposure Routes of Human to Cat Allergens and Sensitization Mechanisms

In humans, exposure to cat allergens (primarily Fel d 1) occurs predominantly through the respiratory tract [73], with secondary routes including conjunctival contact and limited skin exposure [74,75]. Inhalable particles, especially those smaller than 5 μm, can deliver Fel d 1 deep into the lower respiratory airways and represent the main route for asthma induction [76]. In contrast, larger particles are more likely to deposit in the upper respiratory tract and ocular mucosa, commonly leading to symptoms of allergic rhinitis and conjunctivitis [77]. In cases of compromised skin integrity, such as following a cat bite, Fel d 1 may also penetrate the skin and elicit an allergic reaction [78]. The underlying sensitization mechanism involves an exaggerated type 2 immune response [79,80]. Initial mucosal exposure to Fel d 1 leads to allergen capture by antigen-presenting cells (including dendritic cells), which then present the antigen via MHC-II molecules, resulting in T cell activation. Subsequently, activated Th2 cells release key cytokines such as interleukin-4 (IL-4), interleukin-5 (IL-5), interleukin-9 (IL-9), and interleukin-13 (IL-13), that promote the proliferation and differentiation of B cells into antibody-producing plasma cells. These plasma cells produce Fel d 1-specific IgE antibodies, which enter the circulation and bind to FcεRI receptors on the surface of mast cells and basophils, establishing a sensitized state. Upon re-exposure, allergen-induced cross-linking of IgE triggers mast cell degranulation and the release of mediators such as histamine and leukotrienes, ultimately manifesting as clinical symptoms including rhinitis, conjunctivitis, and urticaria [79,81,82,83].

## 3. Indoor Cat Allergen Testing Methods

### 3.1. Immunological Assay

Several immunological methods are used to detect cat allergen proteins, with Fel d 1 detection technologies being the most extensively applied (Table 3). Current technologies, primarily based on the enzyme-linked immunosorbent assay (ELISA) aim not only to enhance sensitivity and specificity but also to address key practical challenges. These include mitigating interference from Fel d 1 polymorphism in quantitative analysis and ensuring that measured results closely correlate with the protein’s in vivo allergenic activity. From rapid screening tools for indoor use to high-precision quantitative standards for manufacturing quality control [84], these approaches provide reliable technical support for accurate allergen management across diverse scenarios. 

### 3.2. High-Throughput Technologies for the Multiplex Detection

To address the need for larger-scale screening of indoor cat allergens (e.g., Fel d 1), a variety of higher-throughput and efficient multiplex platforms have been developed beyond conventional ELISA. Fluorescence-encoded microsphere-based multiplex arrays [91] use spectrally distinct microspheres conjugated with allergen-specific antibodies. These can be simultaneously analyzed using flow-cytometric detection to quantify up to six common indoor allergens [92], including Fel d 1 from a single sample [93]. This technology serves as a powerful tool for epidemiological studies and indoor air quality evaluation, with the potential to be expanded to include other allergens and biologics. Alternatively, molecular techniques that integrate real-time quantitative PCR (qPCR) [94] with electrostatic dust collectors (EDCs) can quantify cat-specific genomic DNA [95] from surfaces, providing an indirect measure of exposure via clothing or other pollutants. Furthermore, high-performance liquid chromatography-tandem mass spectrometry (HPLC-MS/MS) coupled with isobaric tagging enables high-throughput multiplex quantification of allergen extracts and can analyze dendritic-cell responses under allergen challenge [96]. Proteomic approaches have further advanced environmental allergen monitoring: studies by researchers such as Krutz [97] and López-Pedrouso [98] combined LC-MS/MS with bioinformatic pipelines to identify and semi-quantify allergen proteins in complex environmental samples, establishing a powerful tool for systematic profiling of multiple indoor allergens.

### 3.3. Real-Time Monitoring and Emerging Immunosensing Technologies

Bioaerosols consist of airborne particles of biological origin, derived from plants, animals, and microorganisms, that may be either living or dead [99]. These particles are extremely small (ranging from 0.001 to 100 μm in size) and lightweight, allowing them to remain suspended in the atmosphere for extended periods and travel over considerable distances, thereby posing a significant threat to human health [100]. A prominent constituent of bioaerosols is the cat allergen Fel d 1, which is often attached to particles ranging from 2 to 10 μm in size. This allergen can persist in suspension for extended durations and may be repeatedly re-aerosolized by air movement [61]. In recent years, significant advancements have been made in bioaerosol detection technologies, particularly in the fields of immunosensing and non-immunosensing methods [101]. In terms of immunosensing technology, the Quartz Crystal Microbalance (QCM) technique immobilizes Fel d 1-specific monoclonal antibodies via a cystamine self-assembled monolayer; by applying the Sauerbrey mass-frequency relationship, QCM enables label-free detection of airborne cat allergens with a sensitivity reaching the ng/cm^2^ level [102]. Similarly, Surface Acoustic Wave (SAW) immunosensors enhance performance by tracking the slope of phase changes rather than conventional phase shift monitoring, significantly reducing response times and facilitating semi-continuous detection of allergens [103]. Within non-immunosensing technologies, Laser Particle Counters (LPCs) utilize light scattering principles to perform real-time size-resolved particle counting (such as 2.5 μm, 5.0 μm, etc.), thereby allowing indirect estimation of Fel d 1 concentration and spatial distribution [104]. Ultraviolet Laser-Induced Fluorescence (UV-LIF) sensors use targeted UV wavelengths to excite endogenous fluorophores, such as proteins within bioaerosols, and analyze the resulting fluorescence spectra for identification and quantification of biological particles. This makes UV-LIF particularly suitable for continuous, online monitoring of atmospheric bioaerosols [101]. Optical Particle Counters (OPC), also relying on light-scattering mechanisms, classify and count particles based on optical signals generated as they pass through a laser beam. OPC is widely used for cost-effective, long-term monitoring of indoor airborne particles [105]. The emergence of the Internet of Things (IoT) and smart home technologies has further accelerated the integration of multi-sensor networks into indoor environments. These systems are capable of real-time collection of diverse air quality parameters, including carbon monoxide, nitrogen dioxide, and biological contaminants, and transmit the data to central units for analysis. This integrated approach provides a basis for intelligent control of smart ventilation systems, thereby enhancing indoor environmental management [106].

In summary, current cat allergen detection technologies include various methods, each with distinct advantages. Immunoassays such as ELISA remain the gold standard for high-specificity quantification, while high-throughput multiplex platforms (including fluorescence-encoded microsphere arrays and LC-MS/MS-based proteomics) enable efficient, multi-allergen profiling that is used for large-scale studies. Meanwhile, real-time monitoring technologies, such as label-free immunosensors (e.g., QCM, SAW) and optical particle counters (e.g., LPC, OPC), provide dynamic, on-site detection capabilities. The most promising future direction lies in integrating real-time immunosensing with IoT-enabled sensor networks. Such integrated systems would combine the specificity of antibody-based detection with continuous, spatially resolved monitoring, thereby paving the way for smart indoor allergen management systems capable of controlling exposure risks.

## 4. Factors Influencing Indoor Cat Allergen Concentration

### 4.1. Cat-Related Factors

Individual cat characteristics significantly influence the production of Fel d 1. One study shows that age is negatively correlated with skin Fel d 1 levels, with older cats exhibiting markedly lower concentrations than younger ones [107]. Pronounced anatomical variations also exist: facial skin demonstrates significantly higher allergen levels (mean 1015.22 ± 821.6 ng cm^−2^) compared to the thoracic region (mean 115.2 ± 66.8 ng cm^−2^), a trend similarly reflected in hair allergen concentration (face: 63.6 ± 34 µg g^−1^; thorax: 29.6 ± 13.6 µg g^−1^) [46]. Sex is another major factor; combined Fel d 1 concentrations across three trunk sites are higher in males (69.4 mU mL^−1^) than in females (28.9 mU mL^−1^, *p* < 0.05) [44]. This disparity is attributed to sex hormones: neutered males display significantly reduced skin Fel d 1 levels compared to intact males, whereas exogenous testosterone administration restores production to pre-neuter levels [45]. In contrast, among neutered cats, coat color, sex, and hair length exhibit no significant effect on Fel d 1 concentration [43,108].

### 4.2. Environmental and Architectural Factors

Environmental conditions and building characteristics play a critical role in regulating the accumulation and distribution of indoor cat allergens. In households without cats, Fel d 1 levels in settled dust demonstrate a negative correlation with relative humidity [109]. Upholstered furniture—particularly in public areas—acts as a major reservoir for allergens, with both fabric material and usage context markedly affecting Fel d 1 accumulation [67]. Within residential settings, mattress dust presents one of the dominant determinants of overall Fel d 1 concentration. Building age also shows a significant association; dwellings constructed after 1948—particularly those built between 1975–1989 and 1990–2000—show significantly lower Fel d 1 levels in mattresses compared to those built before 1948 (*p* < 0.05) [65].

### 4.3. Human Activity Factors

Human daily activities significantly influence the resuspension and removal of cat allergens. Car seats consistently contain Fel d 1 at concentrations exceeding established sensitization and symptom-provoking thresholds, irrespective of pet ownership status [110], demonstrating the role of human activity in the cross-environmental transport of allergens. Additionally, vacuuming causes an immediate rise in airborne Fel d 1 levels; large particles (10.0–20.0 µm) settle below detection limits, while smaller particles (3.5–6.0 µm) remain suspended for prolonged periods [111]. Even when the vacuum cleaner is turned off, mere mechanical agitation from push/pull motions alone can resuspend large allergen-bearing particles [112]. Vacuum cleaners (VCs) equipped with high-efficiency particulate air (HEPA) filters reduce indoor Fel d 1 levels significantly more effectively than non-HEPA models [113,114]. Furthermore, infrequent washing of bed linens (<1 × week^−1^) and low bedroom ventilation rates (<1 × day^−1^) promote allergen accumulation, reduce dispersal, and indirectly contribute to higher Fel d 1 concentrations [65].

### 4.4. Spatiotemporal Variation Patterns

Fel d 1 concentrations exhibit significant temporal and spatial variability. Seasonal fluctuations are observed, with levels peaking during winter and spring; levels in bed linens during spring can reach up to 2.4-times those recorded in summer [115,116]. In public environments, Fel d 1 accumulates progressively over time, with high-traffic locations exhibiting accelerated accumulation rates. This pattern indicates the role of human foot traffic as a major factor in allergen transport and buildup [117].

## 5. Methods for Preventing and Managing Allergies

### 5.1. Source Control of Allergens

#### 5.1.1. Gene Editing

**Figure 2:**
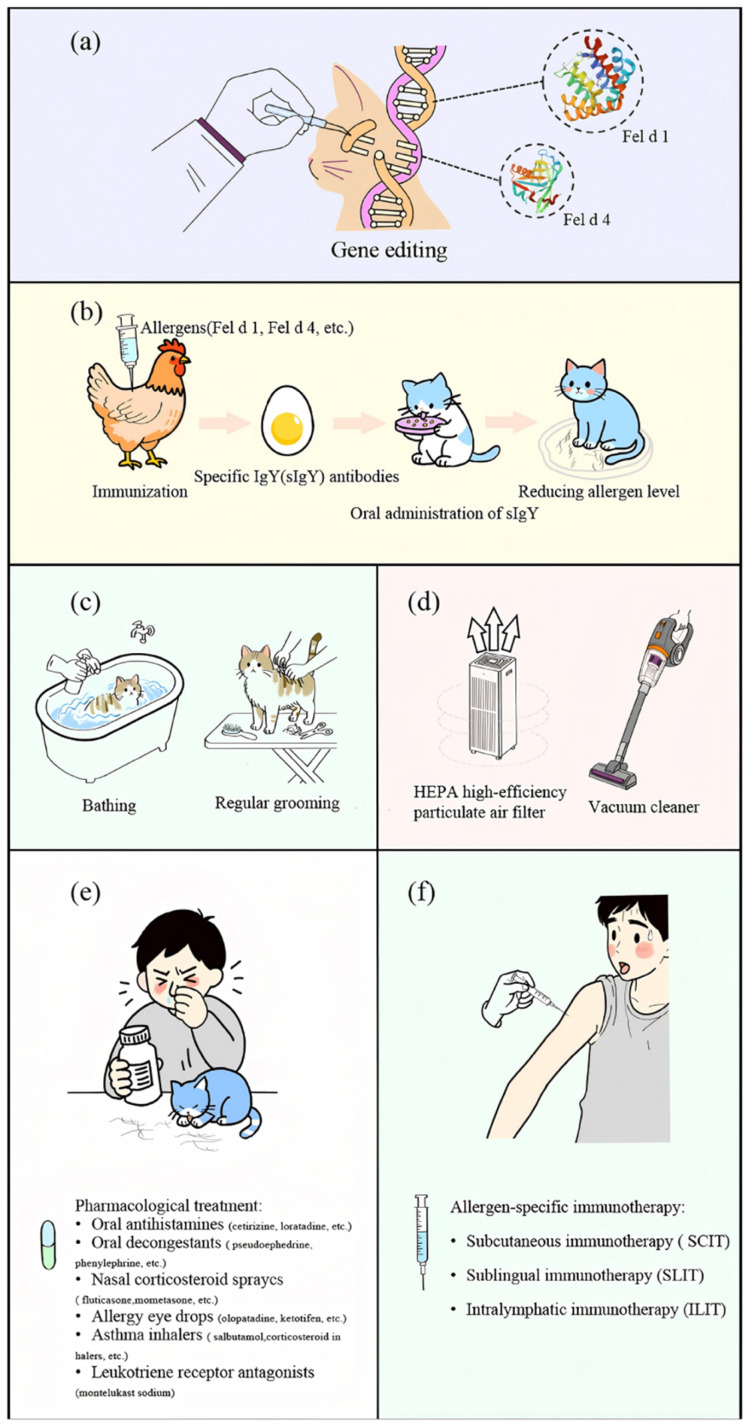
Control of cat allergens. (**a**) Gene editing. (**b**) Immunological methods. (**c**) Physical methods. (**d**) Environmental interventions. (**e**) Chemical drug therapy. (**f**) Immunotherapy.

To achieve long-term allergen reduction, gene editing assays are being developed to generate hypoallergenic cat breeds [118]. Using CRISPR-Cas9, exons CH1 and CH2 were deleted in CRFK cells with an efficiency of 55% [119,120]. Lee et al. generated CH2^−^/^−^ kittens (“Alsik”) via cytoplasmic micro-injection, resulting in a 98.6% reduction in salivary and coat Fel d 1 levels, a trait that was stably maintained through cloning [121]. For immunotherapy, Zhu et al. engineered a human–cat Fcγ–Fel d 1 fusion protein that inhibited IgE-mediated histamine release by over 90% and alleviated airway responses in hFcεRIα mice [122]. Although gene editing presents a promising definitive strategy, potential adverse effects in subsequent generations require further investigation (Figure 2a).

#### 5.1.2. Immunological Methods

Immunoglobulin Y (IgY), a polyclonal antibody produced by egg-laying animals, plays a key role in passive immunity through its ability to specifically bind and neutralize antigens. Following immunization of poultry with specific antigens, the resulting antibodies are transferred from the bloodstream to the ovary and accumulate within the egg yolk [123]. Due to phylogenetic divergence between birds and mammals, IgY exhibits low cross-reactivity within mammalian proteins [124,125]. Satyaraj et al. demonstrated that chicken-derived IgY specifically targeting Fel d 1 effectively inhibits its interaction with IgE [126]. In cat feeding experiments, oral administration of anti-Fel d 1 IgY antibodies led to a 20–47% reduction in salivary Fel d 1 levels [127,128]. Long-term high-dose supplementation over 26 weeks resulted in no adverse changes in body weight, blood biochemical parameters, or toxicological indicators [129]. Under controlled conditions, allergic patients also showed significant improvement in symptoms [130]. Although this method is considered economical, environmentally friendly, and safe, further animal experiments and clinical evidence are required to confirm its efficacy (Figure 2b).

Another strategy involves active immunization of cats. For example, subcutaneous injection of a Fel d 1-CuMV virus-like particle vaccine elicits high titers of neutralizing IgG antibodies in immunized cats [131]. Following vaccination of cats, serum antibody levels increased 300-fold, and the time to develop allergic symptoms in sensitized individuals was delayed 2-fold [132]. In addition, β-lactoglobulin (BLG), a whey milk protein, has been shown to regulate immune responses through the aryl hydrocarbon receptor (AHR) pathway and promote immune cell tolerance [133,134]. Clinical trials indicate that three-month use of lozenges containing BLG micronutrients enhances nasal tolerance to cat allergens in patients [135,136], suggesting that BLG-based intervention strategies may hold promise for preventing allergies not only to cats but also to other furry animals.

#### 5.1.3. Physical Methods

Removing the cat from the household is an effective way to reduce allergen exposure. Studies indicate that indoor airborne Fel d 1 levels decrease by several hundred-fold within 9 to 43 weeks after the removal of the animal [137]. Treatment with sodium hypochlorite solution has been shown to disrupt the structure of recombinant Fel d 1 protein, eliminate its antibody-binding capacity, and inhibit histamine release. However, its practical application is limited due to interference from contaminating proteins, residual toxicity, and corrosive properties [138]. Bathing cats for three minutes using warm water and pet-specific shampoo can reduce airborne allergen levels by 40% to 79% [139,140], though this effect is transient—allergen levels rebound within 3 h and return to baseline within 24 h. Regular grooming (such as brushing and nail trimming) reduces shedding and dander in cats (Figure 2c). Moreover, frequent bathing is often impractical for both feline welfare and owner compliance.

### 5.2. Environmental Interventions

Air filtration can markedly reduce indoor levels of cat allergens (Figure 2d) [141]. Following the use of an air purifier, the Fel d 1 concentration decreased by 76% [142]. However, measurements from nasal samplers indicated a 3- to 5-fold increase in the amount of cat allergen inhaled by individuals. In comparison, high-efficiency particulate air (HEPA)-filtered vacuum cleaners (VCs) demonstrate a more substantial effect on controlling overall cat allergen levels [113], although the vacuuming process temporarily resuspends allergens into the air. In a murine model, photo-electrochemical oxidation (PECO) technology effectively degraded Fel d 1 protein and improved markers of allergic airway inflammation. Nevertheless, this approach remains confined to animal studies and requires further validation [143].

### 5.3. Personal Protection and Medical Intervention

#### 5.3.1. Chemical Drug Therapy

Chemical drug therapy serves as the cornerstone of the symptomatic management for allergic diseases, utilizing various classes of pharmaceuticals that target different pathways of the allergic response (Figure 2e). First-line treatments commonly involve oral second-generation antihistamines, such as cetirizine and loratadine, which alleviate symptoms including rhinorrhea, and pruritus by antagonizing histamine release [144,145]. For nasal congestion, oral decongestants (e.g., pseudoephedrine and phenylephrine) induce vasoconstriction to provide symptomatic relief [146,147]. Intranasal corticosteroid sprays (such as fluticasone [148] and mometasone [149]) are widely recognized as the most effective treatment for allergic rhinitis due to their potent anti-inflammatory effects [150]. Ocular symptoms are managed with topical agents like olopatadine [151] and ketotifen [152]. In allergic asthma, disease management depends on inhaled corticosteroids for sustained control and short-acting beta-agonists [153] (e.g., salbutamol [154]) for acute symptom relief. Furthermore, leukotriene receptor antagonists (e.g., montelukast [155,156]) provide an alternative oral maintenance option by inhibiting inflammatory mediators derived from arachidonic acid metabolism [157,158].

#### 5.3.2. Immunotherapy

Allergen-specific immunotherapy (ASIT) represents an effective treatment for IgE-mediated allergies, including subcutaneous immunotherapy (SCIT), sublingual immunotherapy (SLIT), and intralymphatic immunotherapy (ILIT) (Figure 2f). Through repeated administration of allergens, ASIT elicits antigen-specific immune tolerance [159,160]. The monoclonal antibody regimen REGN1908/1909 demonstrated significant symptom relief and durable efficacy in a phase Ib clinical trial [161]. Omalizumab at a dose of 150–300 mg showed significant clinical benefits and safety in Chinese adult patients with anti-histamine refractory chronic spontaneous urticaria [162]. Another candidate vaccine, PreS-Cat 1-5, is a recombinant fusion protein that uses hepatitis B virus PreS as a carrier for hypoallergenic peptides derived from Fel d 1, Fel d 4 and Fel d 7. This construct elicits potent IgE-blocking antibodies and has shown a favorable safety profile [163]. Nevertheless, ASIT is associated with certain risks: 41% of cat-allergic patients receiving subcutaneous cat allergen extracts experienced severe systemic reactions [164]. Thus, developing novel antigen formulations with high immunogenicity and reduced allergenic toxicity remains crucial for enhancing the safety of ASIT. Additionally, the potential protective effects of early-life pet exposure against asthma-like symptoms appear to be closely linked to family (especially maternal) history of asthma [165,166].

In conclusion, current strategies for managing human allergies to cats include a variety of approaches, ranging from source-directed interventions to environmental controls and patient-level treatments. Source-level approaches, such as gene editing to produce hypoallergenic cats, immunological methods including IgY supplementation and active vaccination of cats, and physical measures like bathing, aim to reduce or neutralize Fel d 1 at its origin. Environmental strategies, including air filtration and vacuuming, lower airborne allergen levels but offer incomplete and often temporary protection. At the patient level, chemical drugs provide symptomatic relief, while immunotherapy seeks to induce long-term immune tolerance. The most promising technologies are those that combine high efficacy with durability and safety. Gene editing, though still in early stages, holds transformative potential for essentially eliminating the allergen source. Next-generation immunotherapies such as engineered vaccines (e.g., PreS-Cat 1-5) and monoclonal antibodies (e.g., REGN1908/1909) that provide improved safety and sustained tolerance induction also represent a major direction for future development. An integrated approach combining selective source control with advanced, personalized immunotherapy may provide the most effective and sustainable solution for cat allergy management.

## 6. Prospects for Future Managing Indoor Cat Allergens

Currently, artificial intelligence (AI) is revolutionizing healthcare by enhancing diagnostics, treatment personalization, and operational efficiency [167]. The integration of immunoinformatics and AI is fundamentally reshaping the development of strategies to prevent and treat human allergy to cat allergens [168,169]. By harnessing advanced computational power to analyze complex biological data—including allergen structures, T-cell and B-cell epitopes, and immune signaling pathways—these technologies enable the precise identification of key therapeutic targets. AI-driven algorithms can predict allergenic epitopes with high accuracy and design hypoallergenic derivatives or multi-epitope vaccines that minimize IgE reactivity while promoting protective immune responses [170]. For example, Zheng et al. optimized the structural linkage between Fel d 1 chains based on AlphaFold2 and identified an optimal linker (2 × GGGGS). The resulting fusion Fel d 1, expressed in *E. coli*, exhibited reduced IgE-binding activity, providing novel insights for molecular diagnosis of cat allergy and designing hypoallergenic vaccines [171]. Furthermore, machine learning models can analyze large-scale clinical and omics datasets to uncover novel biomarkers, stratify patient populations, and guide the creation of personalized immunotherapy regimens [170]. As these in silico predictions are progressively validated through experimental and clinical trials, the synergy of immunoinformatics and AI promises to accelerate the translation of foundational research into safe, effective, and targeted interventions, shifting the paradigm from symptomatic management toward curative immune tolerance.

## 7. Conclusions

Indoor cat allergens trigger human immune disorders such as allergic rhinitis and asthma, posing a threat to individuals. This review provides a comprehensive analysis of cat allergens, including their molecular characteristics, available detection technologies, factors influencing exposure levels, and prevailing control strategies. The dominant allergen Fel d 1 presents a particular challenge due to its complex molecular behavior and persistent presence in the environment, capable of inducing sensitization in the majority of susceptible individuals. Indoor allergen concentrations are regulated by a complex interplay of environmental, architectural, human behavioral, and spatiotemporal factors. Current detection technologies range from specific immunoassays to high-throughput and real-time monitoring platforms. Critically, effective management requires an integrated approach that targets the allergen at its source (e.g., gene editing, oral IgY or immunization), within the environment (e.g., air filtration, PECO technology), and in sensitized individuals (e.g., chemical drugs and immunotherapies). The most promising future direction involves the convergence of these fields, integrating AI and immunoinformatics to design novel hypoallergens, personalize treatments, and incorporate real-time environmental monitoring into smart systems for proactive allergy prevention.

## Figures and Tables

**Table 1:** Frequency of sensitization to cat and dog allergens in different countries [13,20].

Countries	Methods	Number of Subjects Investigated	Cats	Dogs
China	Blood testing	16,664	15.47%	10.50%
Russia	Blood testing	513	24.10%	21.40%
South Korea	Skin prick test	7504	20.60%	15.20%
Germany	Blood testing	356	34.80%	31.70%
Japan	Blood testing	12,205,097	18.20%	18.90%
America	Blood testing	478	54.40%	64.70%
Canada	Skin prick test	623	53.10%	17.30%
Qatar	Skin prick test	473	6.18%	0.50%
Lebanon	Skin prick test	919	29.90%	21.90%
Thailand	Skin prick test	1516	12.90%	10.00%
Nepal	Skin prick test	170	15.30%	14.10%
Mexico	Skin prick test	761	26.70%	33.90%

**Table 2:** Secondary cat allergens and their cross-reactivity characteristics.

Allergens	Protein Molecular Category	Cross-Reactivity	References
Fel d 2	Serum albumin	Can f 3 from dogs	[55,56]
Fel d 3	Cystatin	Can f 8	[49]
Fel d 4	Lipocalin family	Can f 1, Can f 2, Can f 4, and Can f 6 from dogs	[57,58]
Fel d 5	Immunoglobulins	NA	[51]
Fel d 6	Immunoglobulins	NA	[51]
Fel d 7	Lipocalin family	Can f 1, Can f 2, and Can f 4 from dogs	[57]
Fel d 8	Latherin-like protein	NA	[51]

NA: not available.

**Table 3:** Comparison analysis of detection methods for Fel d 1 allergen in indoor environments.

**Methods**	**Technical Features**	**Application Scenarios**	**Sensitivity**	**References**
Signal amplification ELISA	Employing catalytic reported deposition (CARD) technology for the cascade amplification of enzyme-substrate reactions	Ultra-low concentration air samples	156 pg Fel d 1/mL	[85]
Double-point sandwich ELISA	MAbC48 targets conserved IgE epitopes, avoiding interference from Fel d 1 polymorphisms	Indoor dust samples	390 pg Fel d 1/mL	[86]
Immunodot assay (Dustscreen™)	Semi-quantitative, 4 h results, capable of multiple allergen testing	Rapid screening for allergens in public areas	100 pg Fel d 1/mL	[87]
Double antibody sandwich ELISA	Quantified using manufacturer standard curves, between plates CV < 10%	For detecting allergen content in clinical or research settings	80,000 pg Fel d 1	[88]
scFv-Sandwich ELISA	The 96-well high-throughput format replaces traditional radial diffusion, eliminating the need for subjective interpretation	Specific for the potency verification of commercially available standardized allergen extracts	500 pg Fel d 1/mL	[89]
Human IgG4-tweezed sandwich ELISA	Using high-affinity humanized monoclonal antibodies, between plates CV < 9%	Suitable for manufacturers calibration of cat allergen concentrations	250 pg Fel d 1/mL	[90]

## Data Availability

No new data were created or analyzed in this study. Data sharing is not applicable to this article.

## Abbreviations

The following abbreviations are used in this manuscript:

WHO	World Health Organization
IUIS	International Union of Immunological Societies
CRD	component-resolved diagnosis
IL-4	interleukin-4
IL-5	interleukin-5
IL-9	interleukin-9
IL-13	interleukin-13
ELISA	the enzyme-linked immunosorbent assay
CARD	catalytic reported deposition
qPCR	quantitative PCR
EDC	electrostatic dust collectors
HPLC-MS/MS	high-performance liquid chromatography-tandem mass spectrometry
QCM	Quartz Crystal Microbalance
SAW	Surface Acoustic Wave
LPC	Laser Particle Counters
OPC	Optical Particle Counters
IoT	Internet of Things
VC	Vacuum cleaners
IgY	Immunoglobulin Y
BLG	β-lactoglobulin
AHR	aryl hydrocarbon receptor
HEPA	High-efficiency particulate air
PECO	photo-electrochemical oxidation
ASIT	allergen-specific immunotherapy
SCIT	encompassing subcutaneous immunotherapy
SLIT	sublingual immunotherapy
ILIT	intralymphatic immunotherapy
AI	Artificial intelligence
